# Influence of contrast and texture based image modifications on the performance and attention shift of U-Net models for brain tissue segmentation

**DOI:** 10.3389/fnimg.2022.1012639

**Published:** 2022-10-28

**Authors:** Suhang You, Mauricio Reyes

**Affiliations:** Medical Image Analysis Group, ARTORG, Graduate School for Cellular and Biomedical Sciences, University of Bern, Bern, Switzerland

**Keywords:** brain segmentation, pixel attribution, segmentation saliency maps, network interpretability, image augmentation

## Abstract

Contrast and texture modifications applied during training or test-time have recently shown promising results to enhance the generalization performance of deep learning segmentation methods in medical image analysis. However, a deeper understanding of this phenomenon has not been investigated. In this study, we investigated this phenomenon using a controlled experimental setting, using datasets from the Human Connectome Project and a large set of simulated MR protocols, in order to mitigate data confounders and investigate possible explanations as to why model performance changes when applying different levels of contrast and texture-based modifications. Our experiments confirm previous findings regarding the improved performance of models subjected to contrast and texture modifications employed during training and/or testing time, but further show the interplay when these operations are combined, as well as the regimes of model improvement/worsening across scanning parameters. Furthermore, our findings demonstrate a spatial attention shift phenomenon of trained models, occurring for different levels of model performance, and varying in relation to the type of applied image modification.

## 1. Introduction

To date, deep learning has become the state-of-the-art technology to solve problems in medical image analysis (Ker et al., 2017; Litjens et al., 2017; Biswas et al., 2019). However, among the main existing challenges to successfully translate this technology to the clinics, generalization to unseen datasets remains a critical issue (Zhou et al., 2021). Several factors contribute to the issue of model generalization. Among them, one is particularly characteristic of medical imaging applications: protocol variability makes model generalization in medical imaging applications difficult (Glocker et al., 2019). This issue, known as domain shift (Pooch et al., 2019; Stacke et al., 2019; Yan et al., 2019), is an active area of research, and many different approaches and strategies have been proposed in the literature. Among these approaches, they are either applied during the training or testing process. During model training, data augmentation is notably the most popular one, where the objective is to artificially inject variability of intensity patterns, so trained models can cope with unseen variations during testing (Pereira et al., 2016; Liu et al., 2017; Chaitanya et al., 2019; Billot et al., 2020; Sánchez-Peralta et al., 2020). Other approaches applied during training involve a harmonization process that removes protocol-specific patterns (Drozdzal et al., 2018; Delisle et al., 2021; Yu et al., 2021; Zuo et al., 2021). Differently, during test time, proposed methods modify the input test image such that its appearance matches the distribution of a targeted domain (Matsunaga et al., 2017; Jin et al., 2018; Wang et al., 2019), or include a test-time optimization process encoding specific inductive biases known to improve model performance (Wang et al., 2020; Karani et al., 2021). Specifically for Magnetic Resonance (MR) image segmentation, these approaches have focused on data augmentations applied either during training *or* test time, whereas the effects of data augmentation, generally referred hereafter as image modifications, applied during training *and* test time have not been investigated. We postulate this is important since in practical applications performance benefits can be obtained when using both train and test time image modifications. Recently, the work of Sheikh and Schultz (2020) reported interesting results showing that a smoothing operation on training images can lead to improved segmentation performance. However, the limits or regimes of improvement of this type of operation have not been fully studied, as well as approaches that used image modification to achieve better performance during train and test time.

Moreover, the literature has essentially focused on attaining performance improvement using train or test time augmentations. While this is an important objective, we intended to look beyond performance metrics and study the patterns within trained models to further understand why such operations can lead to improved performance. To this end, we turned to interpretability methods to further extract information on models subjected to different combinations of train and test time image modifications.

In order to investigate the effects of image modifications applied during training and test time on model performance, we designed an experimental setup under controlled conditions, constructing a large dataset of 21,000 synthetically generated brain MRI datasets, stemming from 500 real brain images from the Human Connectome Project (Van Essen et al., 2012), and combined with 42 different simulated MR imaging protocols. Through this controlled experimental setup, we aimed at mitigating potential confounder effects, such as the uncontrolled heterogeneity of protocols present on publicly available multi-center datasets, as well as other confounder effects, such as patient-specific variables (e.g., age, gender, etc.) known to potentially bias models (Zhao et al., 2020).

Contrast and texture are two important properties in medical MR images that are dependant on tissue properties and tissue-specific parameters. Early work has shown the importance of texture features as discriminate factors between different tissue types in MRI (Herlidou-Meme et al., 2003). Similarly, Lee et al. (2020) focused on synthesizing different types of endogenous MRI contrast (i.e, T1, T2, etc.) *via* a GAN-based model to achieve similar levels of agreement with radiologists, demonstrating the importance of these two properties for medical diagnostic purposes. In the area of domain-adaptation, a large body of literature also shows the importance of contrast and texture based image modifications. We find methods that explicitly choose contrast or texture based modifications (Agarwal and Mahajan, 2017; Galdran et al., 2017; Sahnoun et al., 2018; Zhang et al., 2019; Sheikh and Schultz, 2020), or use a data-driven optimization approach that select these modifications as the ones yielding the largest effect on model performance (Drozdzal et al., 2018; Wang et al., 2019; Delisle et al., 2021; Karani et al., 2021; Yu et al., 2021; Zuo et al., 2021; Tomar et al., 2022). Recently, Tomar et al. (2022) proposed an optimization-driven image modification approach for test-time domain adaptation where contrast was a dominant image modification found by the approach. In Xu et al. (2020), results on three different datasets showed that contrast and texture modifications have the largest impact on test-time domain adaptation. Xu et al. (2020) also pointed out that compared to global shape features, local textures affect more deep learning networks than human perception.

Therefore, following the observations from the literature, we focused in this study on contrast and texture based modifications to study the spectrum of increased and decreased performance of each type of variation to better characterize and understand where these regimes of improvement occur in trained models. Furthermore, next to analyzing these train and test time regimes, we analyzed how spatial attention of these trained segmentation models changes using interpretability saliency maps (Simonyan et al., 2013; Sundararajan et al., 2017). We adapt in this study interpretability saliency maps to medical image segmentation, in order to investigate the relation between model attention and segmentation performance under the targeted image modification scenarios.

Our experiments show the benefits and interplay when image modifications are applied during training *and* test time, as well as the regimes and patterns of model improvement/worsening for each type of image modification. Furthermore, through interpretability, our findings show a spatial attention shift phenomenon of trained models, occurring for different levels of model performance, and varying with respect to the type of applied image modification.

## 2. Materials and methods

In this section, we first describe how the proposed dataset and experimental design were constructed, followed by detailed descriptions of the two types of targeted image modifications, model training procedure, and evaluation metrics.

### 2.1. Dataset construction

**Figure 1:**
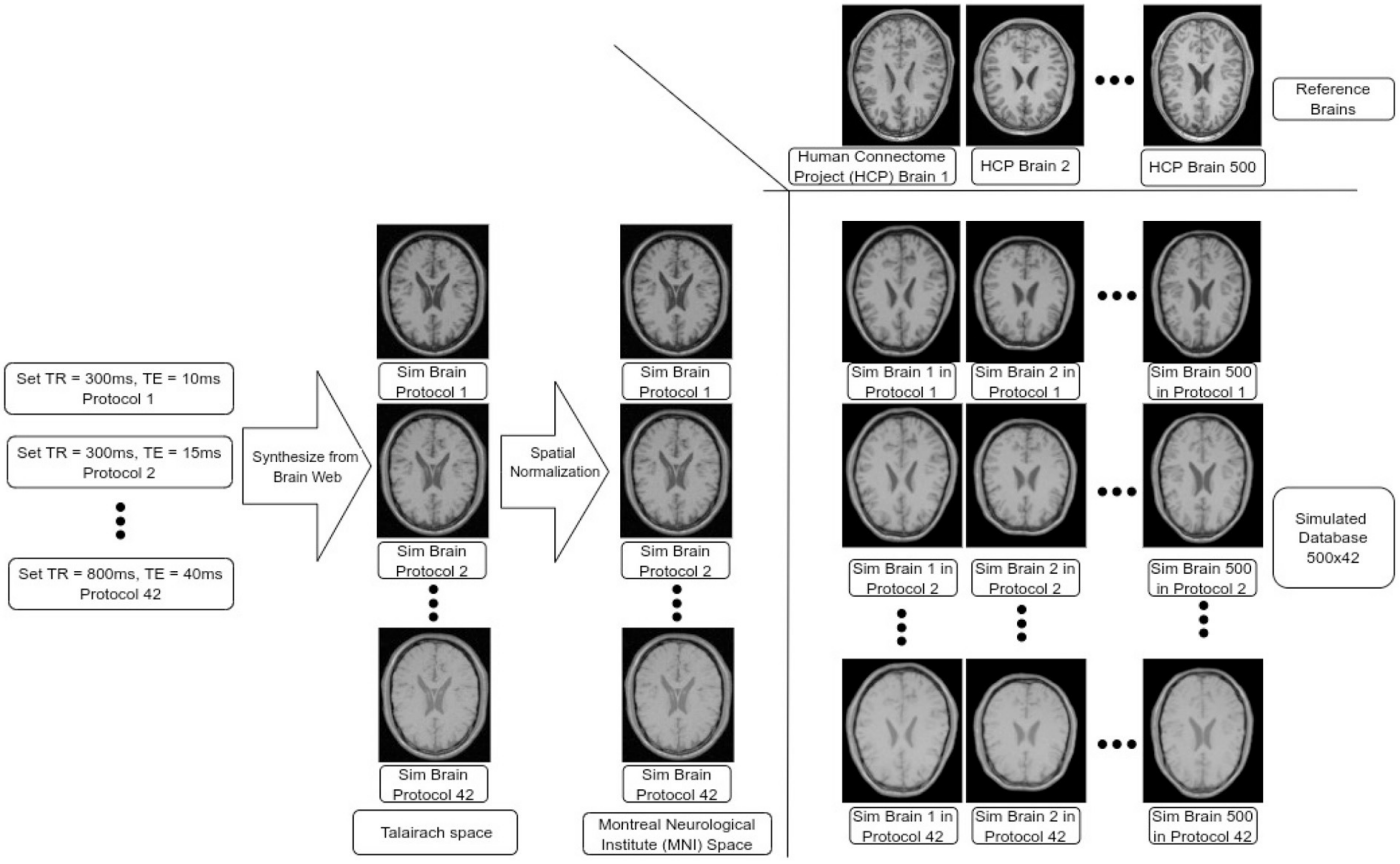
Data-set construction process. We input different pairs of TR and TE values at the customized BrainWeb engine to synthesize brains in Talairach space. We then transformed all these brains into MNI space. Finally, we transformed each simulated brain in MNI space to each of the HCP brains (500 in total) leading to 500 × 42 brains in our data-set.

We constructed a synthetic dataset of brain images simulated across 42 different MR protocols and based on 500 different reference brains from the Human Connectome Project (HCP) (Van Essen et al., 2012), leading to 21,000 simulated brain images, see Figure 1 for an overview of the dataset construction. This construction process is detailed below.

First, simulated BrainWeb (Cocosco et al., 1997) images were downloaded for 42 different protocols. In BrainWeb, custom simulations of normal brain MRI data are based on the anatomical model obtained from the Colin27 brain atlas (Holmes et al., 1998) (the original version in Talairach space) and its fuzzy segmentation of different tissue types. Through BrainWeb, different brain images can be simulated based on modality, scanning technique, slice thickness, flip angle, repetition time (TR), echo time (TE), inversion time (if required by the scanning sequence), and image artifacts including random gaussian noises and intensity non-uniformity fields based on observation in real MR scans. During simulation, we chose to simulate T1-weighted brain images using the spin-echo scanning technique since the major parameters that impact image contrast and texture are TR and TE (Jung and Weigel, 2013). We set 42 combinations of TR and TE pairs, with TR values ranging from 300 to 800 ms with 100 ms intervals, and with TE ranging from 10 to 40 ms, with 5 ms intervals. Other parameters were kept as default. This led to 42 different simulated protocols, which also include labels for Gray Matter (GM), White Matter (WM), and Cerebro-Spinal Fluid (CSF). Each image was then mapped to the Montreal Neurological Institute (MNI) space using a non-linear transformation, described below.

In order to add realistic anatomical variability to the dataset, we employed brain MR images from the HCP. From the HCP dataset, we randomly selected 500 T1-weighted MR images from young adults. Each structural brain image was also non-linearly registered to the MNI-152 brain atlas (Grabner et al., 2006) using Oxford Centre for Functional MRI of the Brain's Non-linear Image Registration Tool (FNIRT) (Jenkinson et al., 2012) produced by the data providers. We used the inverse of these transformations to map each MNI-normalized BrainWeb image to the space of each HCP brain, leading to the final set of 42 × 500 (=21,000) simulated images with corresponding segmentation labels for GM, WM, and CSF. The complete dataset will be also made available for research purposes.

### 2.2. Model training

Apart from texture and contrast image modifications, described below, only z-score normalization was employed as image pre-processing for model training.

In our experiments, we empirically adopted the 4:1 (training vs. testing) split according to the Pareto principle and selected a 16:4:5 split for training, validation, and test sets resulting in 320 training, 80 validation, and 100 testing datasets.

For the training and validation sets, we randomly selected brains from 42 different protocols using a uniform probability distribution to train models under a multi-center configuration, inspired by findings in Hofmanninger et al. (2020). This setup allowed us to assess the impact of image modifications applied during training and test time in a high-throughput manner while avoiding center-specific confounder effects that can occur in practice.

Due to the compute-intensive nature of our experiments, we adopted a standard 2D U-Net architecture (Ronneberger et al., 2015), for which we selected five slices per brain at 10th, 30th, 50th, 70th, and 90th percentile in the cranio-caudal direction to cover the brain anatomy while avoiding selection of empty slices (i.e., only background). Training details are provided below in Section 2.5.

### 2.3. Image intensity modifications: Contrast and texture

Instead of focusing on searching parameters of image modifications leading to optimal performance, as done in previous works, we explored a wide range of positive (i.e., leading to improvements) and negative (i.e, leading to performance decrease) regimes.

**Figure 2:**
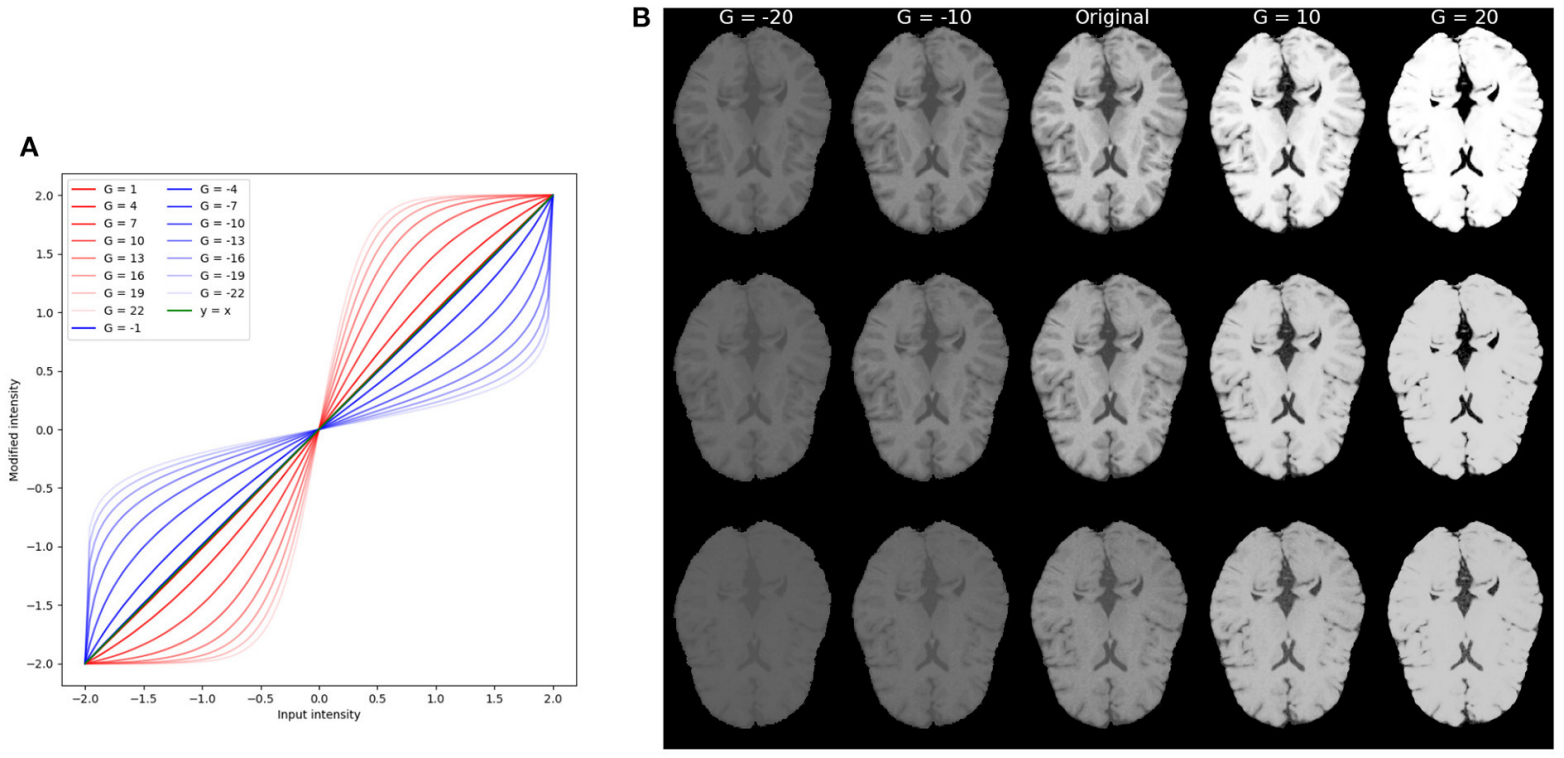
Example of **contrast modifications**. **(A)** Red curves correspond to *G*>0 for increased contrast. Blue curves correspond to *G* < 0, or decreased contrast. The green curve is the identity function. Notice that for |*G*|≤ 1 the filters are visually very close to an equal mapping and overlap on the green curve (identity function) in the figure. **(B)** Example of brain images with contrast modification under different parameter values *G*. The central column corresponds to the original (unmodified) image, and images to its left correspond to decreased contrast. Images to its right correspond to increased contrast. From top to bottom are brain in different protocol settings: TR = 300 ms, TE = 10 ms; TR = 500 ms, TE = 25 ms; TR = 800 ms, and TE = 40 ms.

**Figure 3:**
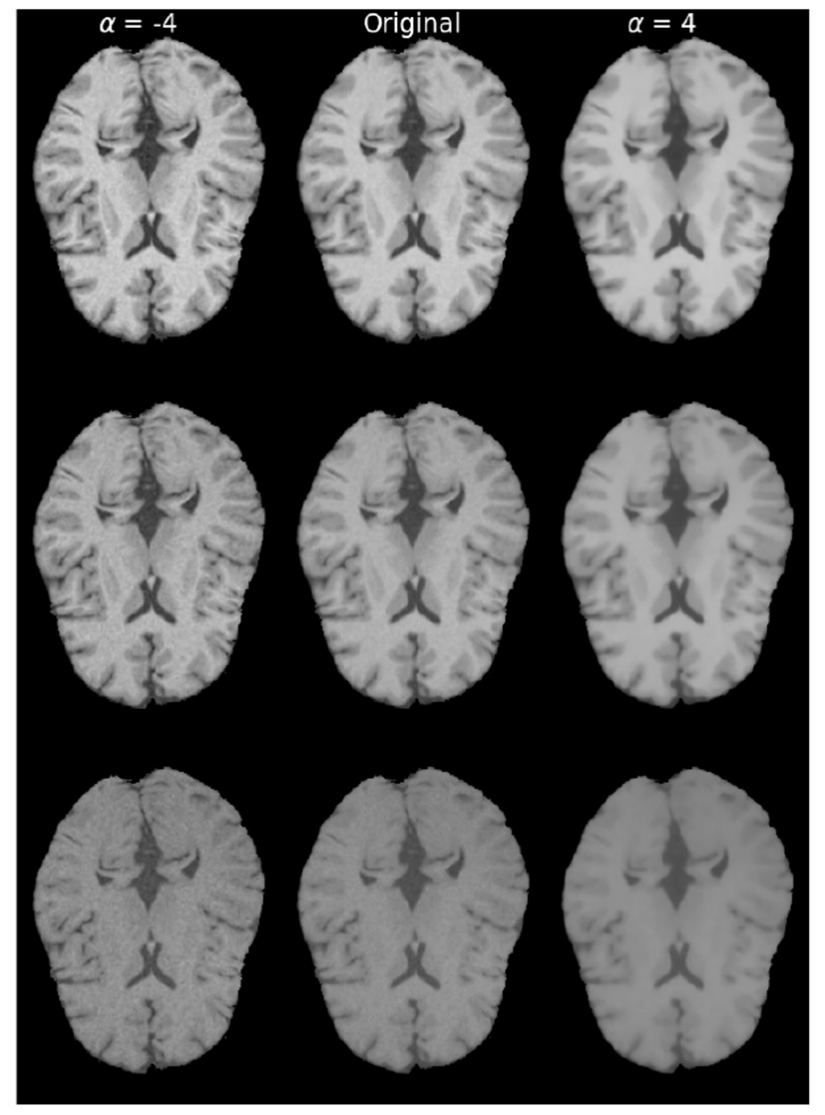
Examples of brain images with different levels of **texture modifications**. Left column with α = −4, displays increased texture, while the rightmost column with α = 4 displays decreased texture. Original denotes the image without any modification. From **top** to **bottom** are brain in different protocol settings: TR = 300 ms, TE = 10 ms; TR = 500 ms, TE = 25 ms; TR = 800 ms, and TE = 40 ms.

For each of the modification we explored a wide range of parameters that drive the modification. Figures 2B, 3 show examples of variations for contrast and texture, respectively. In order to facilitate visualization of increased and decreased effects, throughout the manuscript, we present figures including a central point with performance level for the original image and increased and decreased levels of image modification on each side of this central point.

#### 2.3.1. Contrast modifications

Contrast modification is based on gamma correction, which has been used in previous works to enhance model performance (Wang et al., 2018; Yu et al., 2021). In order to cover the span of negative and positive intensity values, we employed a sigmoidal-logistic filter applied to the foreground of the image being modified. Given an image *f*∈ℝ^*n*^, the contrast modified image *f*_*c*_∈Ω is defined as:


(1) $$\begin{array}{l} f_{c}=\{\begin{array}{ll} \max(f)\cdot \frac{h(\frac{f}{\max(f)}\cdot G)}{h(G)} & G>0\\ \frac{2\max(f)}{G}\ln\frac{\max(f)+f\cdot h(G)}{\max(f)-f\cdot h(G)} & G<0 \end{array},\\ h(G)=\frac{1-\exp(-0.5\cdot G)}{1+\exp(-0.5\cdot G)}, \end{array}$$

where *G*≠0∈ℝ is the gain factor. max(*f*) output the maximum intensity of the input image *f*. When *G*>0, contrast is increased, and when *G* < 0, contrast is decreased. Figure 2A shows examples of contrast-modified images and corresponding sigmoid-logistic modification curves.

#### 2.3.2. Texture modifications

In our study, we chose Total Variation (TV) smoothing for texture modifications, building on the findings of Sheikh and Schultz (2020), where it was shown that TV smoothing leads to a better improved model performance than Gaussian smoothing. To study the opposite (i.e., negative) effect of smoothing, we applied a sharpening filter (Malin, 1977). Below, we briefly describe Total Variation smoothing and sharpening modifications.

TV smoothing is based on image denoising from Rudin et al. (1992). Given an image *f*∈ℝ^*n*^, the smoothed image *u*∈Ω⊂ℝ^*n*^ is found by minimizing,


(2) $$\underset{u\in BV(\Omega )}{\arg\min}\|u{\|}_{TV(\Omega )}+\alpha \int_{\Omega }(f(x)-u(x){)}^{2}dx,$$

where *BV*(Ω) are the bounded variations of domain Ω and the operator $||\cdot |{|}_{TV\left(\Omega \right)}=\underset{\omega }{\int }||\nabla u||dx$ denotes the TV norm of *u*. The TV smoothing parameter α∈ℝ is a weight parameter. In our experiments, we used the split-Bregman-based implementation (Getreuer, 2012) in which a smaller α (α∈ℝ^+^) corresponds to a larger texture modification being applied to the image.

To create the opposite effect of smoothing, we used the sharpening approach from Malin (1977), which creates sharpened images by subtracting the TV smoothed image from 2. The texture-modified image $f_{t}\in \Omega \subset {\mathbb{R}}^{n}$ in our study is defined as:


(3) $$f_{t}=\{\begin{array}{ll} u(\alpha ) & \alpha >0\\ 2\cdot f-u(-\alpha ) & \alpha <0, \end{array}$$

where now α≠0∈ℝ. For α>0 is equivalent to applying Equation (2) and for α <0, the modified image *f*_*t*_'s texture is increased.

#### 2.3.3. Model training schemes under training- and test-time image modifications

Beyond the state of the art focusing on model performance, under image modifications performed either during training or test time, in this study we analyzed the interplay when applying different levels of image modifications. Common knowledge states that optimal model performance would be obtained when the intensity distributions of training and testing images match. We aimed at verifying this expectation, as well as analyzing the regime of improvement and worsening under different levels of image modifications performed during training *and* testing.

For contrast modification experiments, training images were contrast-modified (Equation 1). For one model training trial, the gain factor *G* of the filter was kept equal during training and validation. Empirically, we set *G* ranging from −21 to −2 and from 2 to 21 to study how contrast affects model performance. This range of *G* was selected based on visual assessments to yield a large coverage of modifications. Examples are shown in Figure 2B. This led to 41 different training-time contrast modification settings (including training with unmodified images). Similarly, test images were contrast-modified with the same range of parameters *G*, leading to 41 different test-time image modifications (including unmodified test images). This led to a total of 41 × 41 = 1681 trained models featuring different combinations of contrast-modified images during training and testing (training details presented below in Section 2.5.

Similarly for texture modifications, training images were texture-modified with the filter described in Equation (3) upon feeding the data to the network. For one training trial, the weighting factor α of the filter was the same during training and validation. We set α from −21 to −2 and from 2 to 21 to study the texture enhanced and texture reduced scenarios, which led to 41 different training-time image modification settings including training without any modification. Similarly, for test-time modifications, images were texture-modified, leading to 41 different test-time image modifications including test images without any modification. This range of weighting factor was empirically chosen based on visual assessments to yield a large coverage of modifications. This led to a total of 41 × 41 = 1, 681 trained models featuring different combinations of texture-modified images during training and testing.

For every combination of training and test-time modification, model performance was measured using the dice coefficient for each tissue type and averaged across all 42 pseudo protocols (100 testing images per protocol) to characterize performance for every tissue type. This was performed for contrast and texture based modification experiments.

### 2.4. Interpretability saliency maps for segmentation

Saliency or pixel attribution is a useful tool to analyze relevant pixels for image classification (Simonyan et al., 2013). Integrated Gradient (IG) (Sundararajan et al., 2017) has been widely used in recent research due to its good sensitivity and attribution invariance to model architecture (i.e., given two functionally equivalent models, feature attributions are also equivalent), its implementation simplicity, and efficient computation. In addition, IG not only satisfies the *completeness* property but also has shown to be a metric that captures global non-linear effects and cross-interactions between different features as discussed in Ancona et al. (2017). In our study, we used IG to calculate saliency maps to analyze how spatial attention of trained models changes under different regimes of image modifications. We adapted the original approach proposed for image classification tasks to medical image segmentation. We first describe the original IG approach and then its extension to medical image segmentation.

For a binary CNN classification model *F*:ℝ^*n*^ → [0, 1], the saliency map for a label of input image *x*∈ℝ^*n*^ in the IG method is defined as,


(4) $$IG_{i}^{l}(x)=(x_{i}-x_{i}{}^{\prime })\cdot \int_{\beta =0}^{1}\frac{\partial F^{l}(x^{\prime }-\beta (x-x^{\prime }))}{\partial x_{i}}d\beta ,$$

where *x*′ is the baseline image and *IG*_*i*_(*x*) is the integrated gradients for pixel *i* of input image *x* for class *l*. *F*^*l*^(·)^*mn*^ is the probability at the output of class *l*. β∈[0, 1] is a scalar used for interpolating between the input image (β = 1), and the baseline image (β = 0). Integrated gradients are obtained by accumulating gradients along the path between the baseline image *x*′ and the input image *x*.

To extend IG to segmentation models, we modified IG to integrate gradients from each output pixel to the input image. In order to reduce the computational burden of this task in practice, and benefit from calculations of gradient in deep neuron network platforms, we directly select output tensor to calculate the IG that Tensorflow (Abadi et al., 2015) automatically aggregates the gradients for multiple selections of pixels (i.e., selection of a probability slice for one label instead of a probability pixel for one class). The segmentation IG thus can be denoted as:


(5) $$\begin{array}{l} IG_{i}^{l}(x)=\sum_{m=1}^{M}\sum_{n=1}^{N}IG_{i}^{l}{\left(x\right)}^{mn}\\ \;=\sum_{m=1}^{M}\sum_{n=1}^{N}\left(x-x^{\prime }\right)\cdot \int_{\beta =0}^{1}\frac{\partial F^{l}{\left(x^{\prime }-\beta (x-x^{\prime })\right)}^{mn}}{\partial x_{i}}d\beta \\ \;=(x-x^{\prime })\cdot \int_{\beta =0}^{1}\frac{\sum_{m=1}^{M}\sum_{n=1}^{N}\partial F^{l}{\left(x^{\prime }-\beta (x-x^{\prime })\right)}^{mn}}{\partial x_{i}}d\beta \\ \;=(x-x^{\prime })\cdot \int_{\beta =0}^{1}\frac{\partial F^{l}(x^{\prime }-\beta (x-x^{\prime }))}{\partial x_{i}}d\beta \end{array}$$

The term $IG_{i}^{l}\left(x\right)$ is the integrated gradients of the label *l* for input pixel *i*, and $IG_{i}^{l}{\left(x\right)}^{mn}$ corresponds to the integrated gradients of the input pixel *i* for the output that indexed *mn* in the label *l*. The operator · denotes element-wise multiplication. *F*^*l*^(·)^*mn*^ is the probability at the output indexed *mn* for label *l*, which we omit in the equation for clarity. *M* and *N* are the size in pixels of the input image *x* that *M* = *N* = 288.

In our experiments, we calculated saliency maps according to each of the labels of interest, i.e., CSF, Gray Matter, and White Matter. To implement Equation (5), we used a trapezoidal-based interpolation, using $\Delta \beta =\frac{1}{16}$ as a trade-off between accuracy and calculation time and GPU memory. In order to normalize pixel attribution values so they are not affected by tissue intensities (i.e, high intensity pixels having larger attribution), we re-scaled IG gradients using the 5% and 95% percentile of calculated IG values to −1 and 1.

Due to the compute-intensive nature of IG for segmentation (Equation 5 for all the brains and protocols we simulated, we randomly selected a subset of brain images from a representative protocol with settings TR = 500 ms and TE = 25 ms, and calculated saliency maps of each label on cases yielding the best, worst performances, and the original (unmodified) case for comparison purposes. We chose this protocol as a representative one since it is situated in the center of all simulated protocol parameter values. Calculated saliency maps for each type of image modification were then averaged across trained models for each label and selected slice.

### 2.5. Implementation details

We used the U-Net architecture (Ronneberger et al., 2015) and modified its output to multi-class segmentation to segment CSF, GM, and WM. We modified the input size to 288x288 based on the largest size of the bounding box of all slices in the training and testing set. We also added a dropout layer before each pooling layer. As loss function, we calculated the mean cross-entropy of each output class for each input batch.

Trained models were saved at the end of each epoch and were evaluated *via* the corresponding validation set. We selected models with the lowest validation loss among all training epochs of that trial. Models were trained with 250 epochs. We operated all experiments on Tensorflow (Abadi et al., 2015) 2.4. To reduce stochasticity during training and to make a fair comparison across trained models we implemented three strategies: (i) all training trials used the same initialization and seed, (ii) we set training to the deterministic operation mode in Tensorflow (Abadi et al., 2015), and (iii) we used the same training data but used different random shuffles during training, and performance across all 20 runs (one run evaluated on 42 × 100 cases) was then averaged for analysis purposes. We used Adam optimizer with a learning rate of 1e-4 during training and set the dropout rate to 0.5 for generalization purposes. We used GeForce GTX 1080Ti GPUs during experiments. The synthetic data repository and the code to calculate integrated gradients in segmentation models will be made available.

## 3. Results

In this section, we describe the main results divided into (i) effect of contrast modifications on performance of trained models, (ii) effect of texture modifications on performance of trained models, and (iii) interpretability analysis of U-Net's spatial attention levels for different regimes of contrast and texture modifications. The first two experiments (i) and (ii) aim at analyzing the interplay when applying different levels of image modifications. Particularly, these two experiments also aim at verifying whether optimal performance occurs when the intensity distribution of training and testing images match. Furthermore, these two experiments aim at analyzing the regime of improvement and worsening under different combinations of image modifications performed during training and testing. The third experiment, on the interpretability of the U-Net's spatial attention, aims at analyzing how spatial attention of trained models changes under different regimes of image modifications, and their application during training and test time.

### 3.1. Effect of contrast modifications

**Figure 4:**
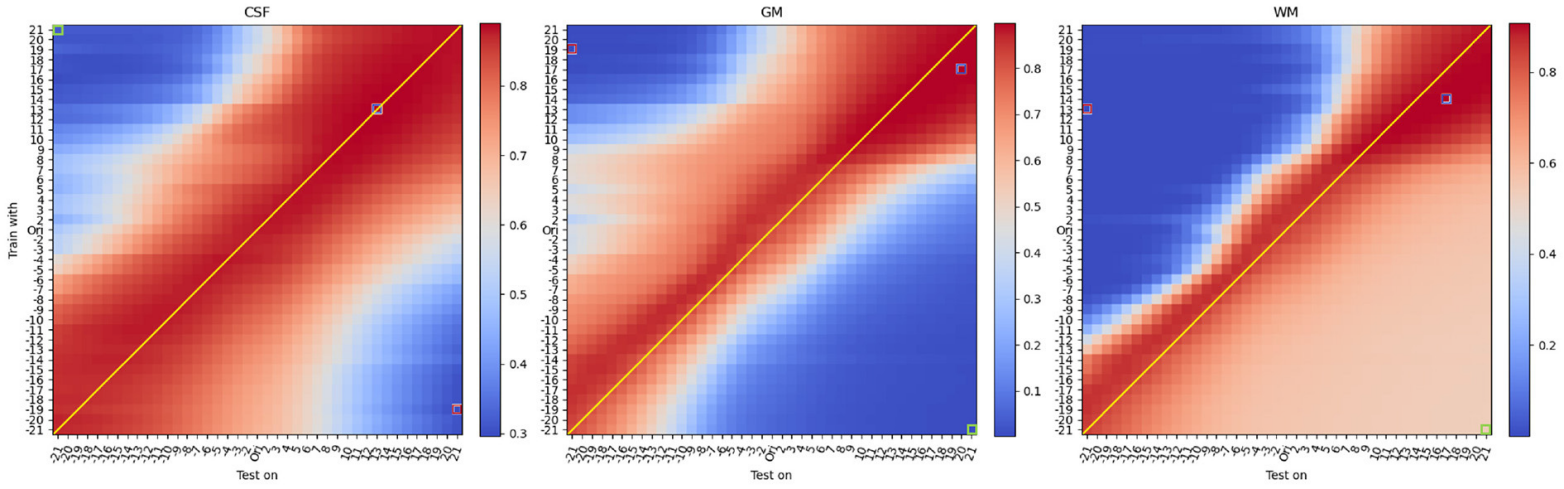
Mean DSC of all protocols under different **contrast modifications**. From **left** to **right**, each heatmap denotes DSC performance for CSF, Gray Matter, and White Matter. On each heatmap, the vertical axis denotes different levels of contrast modification, with parameter *G* ranging from −20 to 2 and from 2 to 20, to *train* models. In the vertical axis, “Ori” denotes models trained with original (unmodified) training images. The horizontal axis denotes different levels of contrast modification, ranging from −21 to 2 and from 2 to 21, to *test* models. Similarly, “Ori” in the horizontal axis denotes models tested on original (unmodified) images. For *G* < 0, the contrast decreases and for *G*>0, the contrast increases. DSCs of the best and worst, and another exemplary bad model performance, opposite to worst in terms of the combination of modifications, are labeled with red, blue, and green frames, respectively. Interestingly, and differently from what one would expect, best performance is not occurring when there is a matching between train and test time modification (i.e., anti-diagonal). See Table 1 for details.

Figure 4 shows results of the mean DSC across all 42 protocols under different contrast modifications, for a total of 42 × 41 × 41 = 72, 324 evaluations, which are summarized as grid points on Figure 4 (see Supplementary material for an animated version including saliency maps).

From the mean DSC heatmap, we first noticed that the best DSC performance did not occur for models trained and tested on unmodified data (i.e., central point in Figure 4). We observed that optimal performance occurs for contrast modifications applied during training and testing. We also observed that optimal performance did not occur on matching intensity distributions (i.e., indicated with a thin line in Figure 4), but an upward shift of the anti-diagonal was observed for models yielding improved DSC values across all three tissue types. The upward shift impact can be also observed in **Figure 6**, where for the unmodified pair (original), the U-net tends to yield an under-segmentation of GM and over-segmentation of WM, leading to sub-optimal performance across the column denoted 'Ori' in Figure 4.

Considering the specific scenario where contrast modifications are only applied either during training (i.e., central columns on each heatmap of Figure 4) or only during test-time (i.e., central rows on the heatmap of Figure 4), results show that contrast modifications can boost model performance. Moreover, combining these two scenarios, to perform contrast modification during training and test-time, the best performance across all configurations could be found, as indicated by the blue-framed squares in Figure 4. Details of DSC values are shown in Table 1. Concerning the central point of reference, the best performance corresponds to an up to 10% performance improvement for GM, 8% for WM, and 1.5% for CSF. However, improvements cannot be attained by continuously increasing contrast, as shown in Figure 4.

**Table 1:** Mean and standard deviation of best models achieved in the settings of contrast and texture modifications to the original settings (unmodified).

	**CSF**	**Gray matter**	**White matter**
Original	0.877 ± 0.007	0.813 ± 0.032	0.841 ± 0.026
Contrast modification best	0.89(↑1.5%)±0.005	0.897(↑10%)±0.003	0.908(↑8%)±0.005
Texture modification best	0.881(↑0.5%)±0.006	0.839(↑3%)±0.024	0.859(↑2%)±0.019

**Figure 5:**
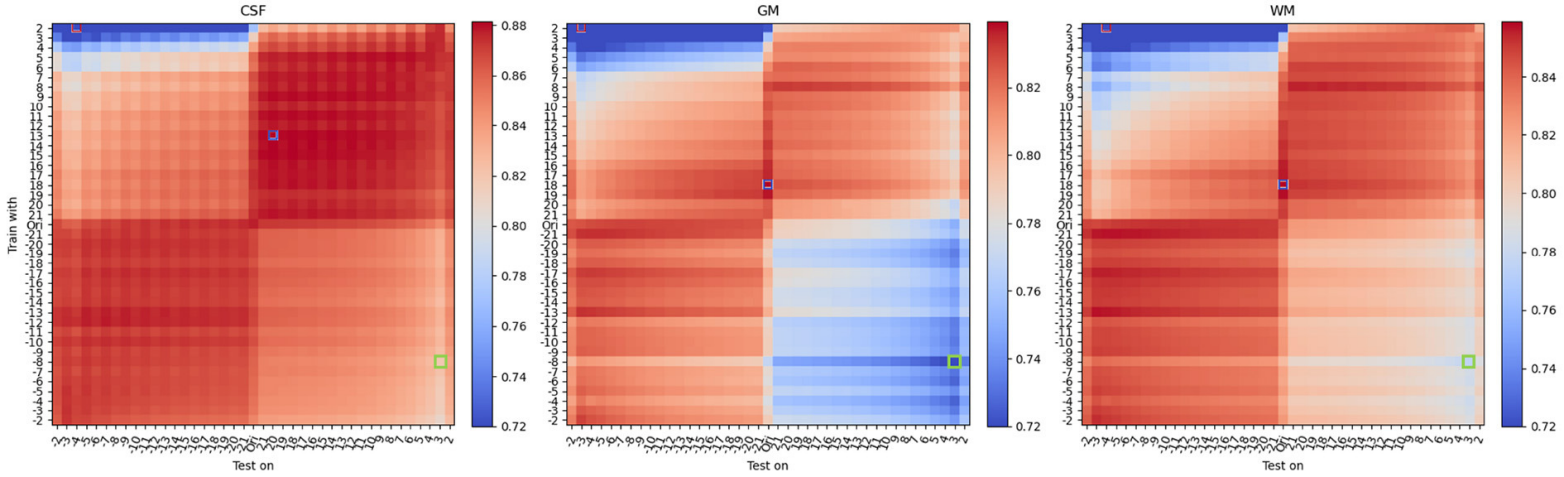
Mean DSC of all protocols under different **texture modifications**. From **left** to **right**, each heatmap denotes DSC performance for CSF, Gray Matter, and White Matter. On each heatmap, the vertical axis denotes different levels of texture modification, ranging from −4 to −20 and from 20 to 4, to train models. In the vertical axis, “Ori” denotes models trained with original (unmodified) training images. The horizontal axis denotes different levels of texture modification, ranging from −4 to −20 and from 20 to 4, to test models. Similarly, “Ori” in the horizontal axis denotes models tested on original (unmodified) images. *Note that in texture modification, smaller* |α| *corresponds to smaller modification effect thus the reversed order in indexing*. For α <0, the texture is enhanced (sharpened) and for α>0, the texture is reduced (smoothed). DSCs of the best and worst, and another exemplary bad model performance, opposite to worst in terms of the combination of modifications, are labeled with red, blue, and green frames, respectively. Interestingly, and differently from what one would expect, best performance is not occurring when there is a matching between train and test time modification (i.e., anti-diagonal). See Table 1 for details.

The two “best” results correspond to the blue framed grid points in Figures 4, 5. ↑ describes relative improvements with respect to results on the original images. Interestingly, and differently from what one would expect, best performance is not occurring when there is a matching between train and test time modification (see anti-diagonal on Figures 4, 5).

These results show the importance of considering both training and test time modifications to boost model performance.

At the top right corner of the heatmaps in Figure 4, we observe a broadening of the region where improvement occurs, mostly noticeable for WM. When increasing the contrast of the training and testing images, the intensity difference among tissues increases, resulting in a larger area of performance improvement (shown as a slightly broader red-colored area in Figure 4).

**Figure 6:**
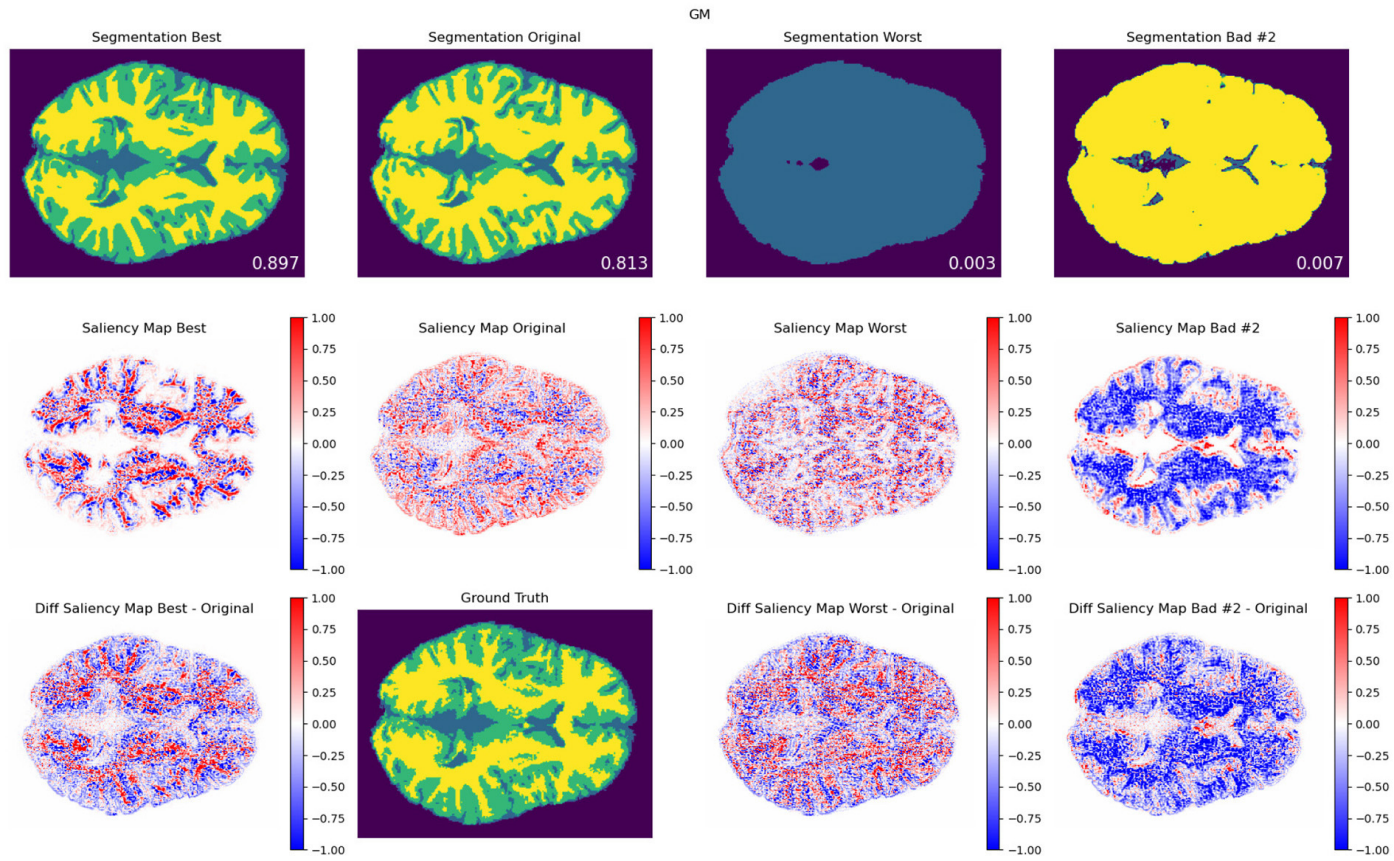
**Contrast modifications**. GM segmentation results **(top)**, saliency maps **(middle)**, and saliency difference maps **(bottom)**, for best (first column), original (second column), worst (third column), and another exemplary bad model performance (fourth column) opposite to worst in terms of the combination of modifications, for protocol TR = 500 ms; TE = 25 ms. The best, worst, and second bad cases corresponded to the blue, red-, and green-framed squares on heatmaps in Figure 4. As a reference, the DSC values for best, original, worst, and other bad result models are included for each segmentation result that are averaged among 20 trained models with the same modification setting pair. An attention shift or redistribution of pixel attribution is observed between the original, best, and worst performances. Compared to the ground truth, the unmodified pair (original) tends to have an under-segmentation of GM and over-segmentation of WM.

**Figure 7:**
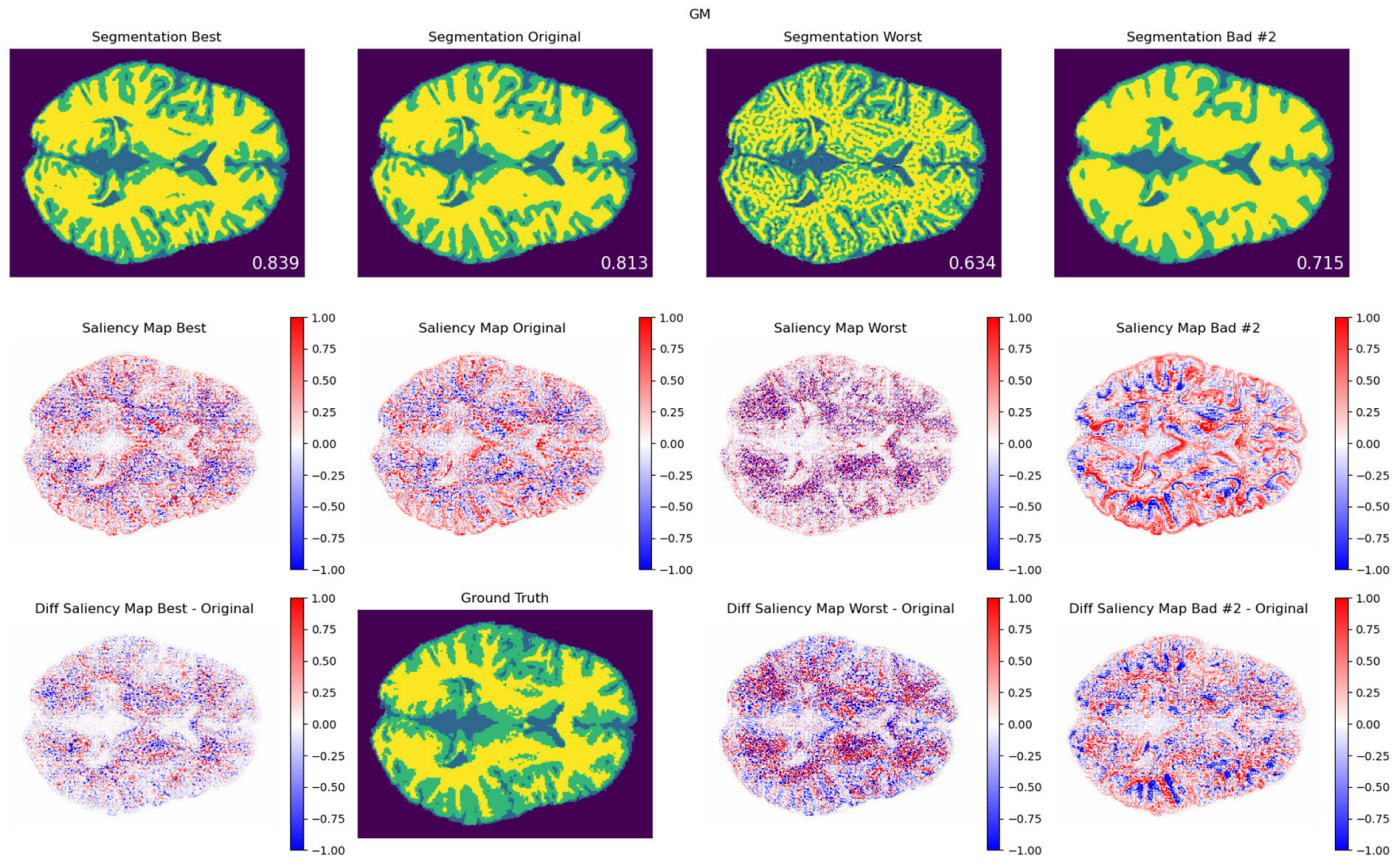
**Texture modifications**. GM segmentation results **(top)**, saliency maps **(middle)**, and saliency difference maps **(bottom)**, for best (left column), original (middle column), worst (third column), and another exemplary bad model performance opposite to worst in terms of the combination of modifications, for protocol TR = 500 ms; TE = 25 ms. The best, worst, and second bad cases corresponded to the blue, red-, and green-framed squares on heatmaps in Figure 4. As a reference, the DSC values for best, original, worst, and other bad result models are also included. An attention shift or redistribution of pixel attribution is observed among the different performances. Compared to the ground truth, the unmodified pair (original) tends to have an under-segmentation of GM and over-segmentation of WM.

For contrast-based modifications, we observed that sub-optimal results occur when different directions of modifications are used (e.g., increase contrast during training and decreased contrast during testing). This is illustrated on the main diagonals in Figures 4, 5. We also observed that the region of performance improvement is different for different tissue types. For GM, the performance drops more rapidly than for WM in the regions where contrast is decreased during training and increased during test time. This is caused by an under-segmentation of GM and an over-segmentation of WM, as shown in Figures 6, 7, top rows. Conversely, in the top-left regions of Figure 4, where contrast is increased during training but decreased during test time, WM is under-segmented and its DSC value drastically drops to values close to 0. As we approach the top-left corner of the heatmaps, WM is first falsely predicted as GM. However, when further approaching the top-left corner, both tissues are falsely predicted as CSF or background.

### 3.2. Effect of texture modifications

Figure 5 shows results of the mean DSC across all 42 protocols under different texture modifications, for a total of 42 × 41 × 41 = 72, 324 evaluations, which are summarized as grid points on Figure 5. Performance improvements for texture-based modifications are summarized in Table 1. In comparison to contrast, texture-based modifications yielded in average a lower level of performance improvement of 3% (GM), 2% (WM), and 0.5% (CSF) with respect to models trained on original (unmodified) images.

Similarly as for the contrast modification experiments presented above, for texture modification we found that the best DSC did not occur for models trained and tested on unmodified images. However, compared to the contrast modification experiments, we found a different pattern of performance improvement and worsening. As shown in Figure 5, the best performance was found for images modified during training, using a negative texture α parameter value (i.e., smoothing). This aligns with the findings from Sheikh and Schultz (2020) and further suggests that no major benefits occur when images are texture-modified during test-time.

From Figure 5, we also observed a cross-like pattern, not observed for contrast-based modifications. This is due to the non-linear effect of the parameter α on the optimization process of Equation (2). These results suggest that in comparison to contrast-based modifications, texture-based modifications have a more unstable behavior. This is for instance seen at several areas in Figure 5 presenting non-monotonic patterns of performance change. In terms of tissue types, CSF showed a similar level of benefit to contrast-based modifications, while contrast modifications yielded larger levels of improvement for GM and WM than texture-based modifications, as shown in Table 1.

### 3.3. Interpretability analysis of U-Net's spatial attention levels for different regimes of contrast and texture modifications

In Figures 6, 7, we show examples of GM segmentation results and corresponding saliency maps for best and worst performance for protocol with TR = 500 ms and TE = 25 ms of contrast modification and texture modification. As a reference, we also show segmentation results and corresponding saliency maps for the original (unmodified) image. The segmentation results are averaged among 20 trained models for one parameter setting. Additionally, following the findings from Figures 4, 5, where we observed two areas of performance worsening (top-left and bottom-right quadrants), we complemented Figure 6 with another example of bad performance, opposite to the position of the worst performance in terms of the combination of modifications. This was performed in order to further investigate how the model's attention changes under opposite schemes of image modifications.

In terms of saliency maps for contrast-based modifications (Figure 6), we observed a general attention shift of trained models under different image modifications. Particularly, we observed that such attention shift seems to be related to spatial redistribution of attended areas: for GM segmentation, the best performance models yielded a more concentrated spatial distribution to the edgy area between GM and WM, whereas worst and the second bad performance models tend to falsely yield areas of increased attention to the unrelated area, e.g., the inner area of white matter. We also noticed that there is an 'inverted' value change of the saliency maps between GM and WM under the same parameter setting, shown in Figure 6 and the Supplementary materials.

For texture-based modifications, similarly as for contrast, we observed performance improvements when trained models shifted their attention (see Supplementary material). Similar to contrast, such an attention shift seems to enhance the attention to attended areas. However, since the performance change is relatively small compared to contrast experiments, the effect is less strong. Strikingly, a shift occurs toward edge areas of tissues, which might be related to the nature of texture modifications mostly affecting edge areas. We also observe the 'inverted' or complementary pattern of saliency maps which relate to the miss-segmentation between two tissues. In Supplementary material, we include several other brain cases showing similar results of the found attention shift phenomenon. In the next section, we discuss and summarize our results in light of the state of the art and include limitations and future work.

## 4. Discussion

Model generalization is a crucial aspect of training deep learning models. In these regards, domain shift stemming from differences in protocols is one of the most difficult problems negatively affecting model generalization. Among the most successful approaches proposed to ensure model generalization, methods modifying intensity patterns during training or test time have shown promising results (Liu et al., 2017; Matsunaga et al., 2017; Drozdzal et al., 2018; Jin et al., 2018; Chaitanya et al., 2019; Wang et al., 2019, 2020; Billot et al., 2020; Sánchez-Peralta et al., 2020; Sheikh and Schultz, 2020; Delisle et al., 2021; Karani et al., 2021; Yu et al., 2021; Zuo et al., 2021), with contrast- and texture-based modifications being the most impactful image modifications applied either explicitly (i.e., direct modification) or implicitly (i.e., *via* a data-driven pipeline).

Differently from the state of the art, mainly focusing on performance objectives, in this study we aimed at further analyzing and leveraging our understanding as to how and to which extent, these two types of image modifications affect the performance of trained models, when applied during training and/or test time. Furthermore, beyond performance metrics, we believe interpretability can play an important role in leveraging the generalization capability of models for medical applications. Toward these objectives, in this study, we designed a controlled experiment, consisting of a large synthetic dataset of 21,000 brain MR images based on 500 datasets from the Human Connectome Project, and 42 different MR protocols, designed in relation to the major parameters impacting image contrast and texture in MRI (Jung and Weigel, 2013).

Overall, our study highlights the benefits of utilizing contrast and texture-based modifications for improved performance, with contrast-based modifications yielding larger performance improvements than texture-based modifications. This finding aligns with recent data-driven approaches, wherein an optimization process modifies images till the best performance is attained, resulting in images characterized by a contrast change (Drozdzal et al., 2018; Delisle et al., 2021; Yu et al., 2021) (see Figure 2 middle in Delisle et al. (2021) as a notable example of this). We note that this strategy has proved successful for both training and test-time modifications, but has not been analyzed in conjunction, as done in this study, which has shown the benefits of combining them during training and test time. These results and findings also contribute to a more general discussion regarding the design of image acquisition protocols, that historically have been fine-tuned for human perception, but might not necessarily be optimal for deep learning models, as also hinted in the study of Delisle et al. (2021).

The backbone of our experiments is a large dataset of synthetically generated MR brain images, designed to train and test an extensive set of segmentation models utilizing various simulated MR protocols, and contrast and texture-based modifications. Despite the synthetic nature of this dataset and the related disadvantages of not using a real one, we believe that the advantages of using this dataset for the objectives of this study are superior and outweigh the utilization of a real dataset wherein different confounder effects could bias our analyses. While many publicly multi-protocol datasets are available for research purposes, most of them do not fully characterize protocol variability, demographics, etc., or lack important information known to cause generalization problems. Conversely, other available datasets have been designed for specific research questions and imaging protocols, hence limiting analyses of generalization capability in clinical scenarios. Hence, our interest to design a controlled and high-throughput experiment. In addition, the generated dataset also simulates a large set of brains being scanned over 42 simulated MR protocols, which we think can be considered for studies where anatomical variability is relevant. Beyond this study, we believe that this dataset can be useful in other areas of research, such as in federated learning where typical data imbalances naturally occur, and related confounder effects have been pointed out as one of the issues to be solved (Aledhari et al., 2020; Balachandar et al., 2020; Willemink et al., 2020; Qu et al., 2021). As part of this study, we will share the complete dataset, along with accompanying parametric information for research purposes.

Interpretability of deep learning has attracted much attention in the medical image computing community (Cardoso et al., 2020; Reyes et al., 2020; Budd et al., 2021; Fuhrman et al., 2021; Kitamura and Marques, 2021; McCrindle et al., 2021). The so-called “black box” nature of deep learning networks, in conjunction with issues of shortcut learning (Geirhos et al., 2020), confounding effects (Zhao et al., 2020), and other critical issues in the training of deep learning models, further exacerbate the need to develop interpretability approaches allowing developers and end-users of these technologies to audit them and gain insights on their patterns of functioning. In this study, we extended the approach of integrated gradients (Sundararajan et al., 2017), designed for classification tasks, for multi-class segmentation tasks. However, we acknowledge that other algorithms for saliency calculation might be extended for segmentation tasks in different approaches. Results of this analysis showed an interesting phenomenon, up to our knowledge not previously analyzed in detail, where an attention shift occurs as a function of the type of image modification being used, and follows distinctive patterns for increased and decreased performance levels. Indeed, although attention mechanisms have attracted much popularity to improve model performance (Chaudhari et al., 2021), we believe that gaining more understanding of these patterns might open new opportunities to use them as model fingerprints to detect failure modes, enhance training monitoring, improve quality control of training datasets, etc.

Some limitations are worth mentioning. The study focused on analyses for brain MRI imaging studies. Further work is needed to verify these findings apply to other medical scenarios. Our analysis in this study remains qualitative *via* visualizations of saliency maps across different brain datasets. In this regard, further research work is needed to design quantification metrics for the observed attention shift phenomenon. An interesting avenue of research in these regards concerns the use of these model's fingerprints to guide quality assurance of models in a similar way as it has done before where segmentation outputs have been used to predict model performance (Kohlberger et al., 2012; Robinson et al., 2018; Hann et al., 2019; Liu et al., 2019). The study focused on image modifications based on contrast and texture, which are popular image modifications used in the literature. Further research is needed to verify how the observed attention shift reported here occurs for other types of image modifications.

## 5. Conclusion

In this work, we performed a high-throughput analysis of contrast- and texture-based modifications applied during training and test-time of deep learning models, using a controlled experimental setting employing datasets from the Human Connectome Project and a large set of simulated MR protocols, in order to mitigate the inhomogeneity of data confounders, and investigate possible explanations as to why model performance changes when different levels of contrast and texture-based modifications are used. Our experiments confirm previous findings regarding the improved performance of models subjected to contrast and texture modifications employed during training and/or testing time, but further show the interplay when these operations are combined, as well as the regimes of model improvement/worsening across scanning parameters. Furthermore, our findings demonstrate a spatial attention shift phenomenon of trained models, occurring for different levels of model performance, and varying in relation to the type of applied image modification. We expect these findings and data resources to further leverage the generalization capability and understanding of trained deep learning models for clinical applications.

## Data availability statement

The raw data supporting the conclusions of this article will be made available by the authors, without undue reservation.

## Author contributions

SY and MR contributed to conception and design of the study. SY organized and executed the dataset, experiments, analysis of results, and wrote the first draft of the manuscript. Both authors contributed to manuscript revision, read, and approved the submitted version.

## Funding

This document was the results of the research project funded by Swiss Personalized Health Network (SPHN) initiative and supported by the Swiss National Science Foundation under grant number CRSII5_180365 (The Swiss-First Study).

## Conflict of interest

The authors declare that the research was conducted in the absence of any commercial or financial relationships that could be construed as a potential conflict of interest.

## Publisher's note

All claims expressed in this article are solely those of the authors and do not necessarily represent those of their affiliated organizations, or those of the publisher, the editors and the reviewers. Any product that may be evaluated in this article, or claim that may be made by its manufacturer, is not guaranteed or endorsed by the publisher.
