# Supplementary material for: Influence of contrast and texture based image modifications on the performance and attention shift of U-Net models for brain tissue segmentation
Source: Front Neuroimaging. 2022 Oct 28;1:1012639. doi: 10.3389/fnimg.2022.1012639 (PMC10406260; doi:10.3389/fnimg.2022.1012639)
Supplement: Supplementary file 1 [file Data_Sheet_1.PDF]

Supplementary Material of Influence of Contrast and Texture based Image Modifications on the Performance and Attention Shift of U-Net Models for Brain Tissue Segmentation

EXAMPLE SLICES OF SALIENCY MAPS OF GM AND WM IN CONTRAST MODIFICATION.

Shown in Fig. S1 and Fig. S2, WM and CSF segmentation results (top row), saliency maps (middle row), and saliency difference maps (bottom row), for best (first column), original (second column), worst (third column), and another exemplary bad model performance (fourth column) opposite to worst in terms of the combination of modifications, for vendor TR = 500 ms; TE = 25 ms. The best, worst, and second bad cases corresponded to the blue, red-, and green-framed squares on heatmaps in Fig. 4. As a reference, the DSC values for best, original, worst, and other bad result models are included for each segmentation result. An attention shift or redistribution of pixel attribution is observed between the original, best, and worst performances.

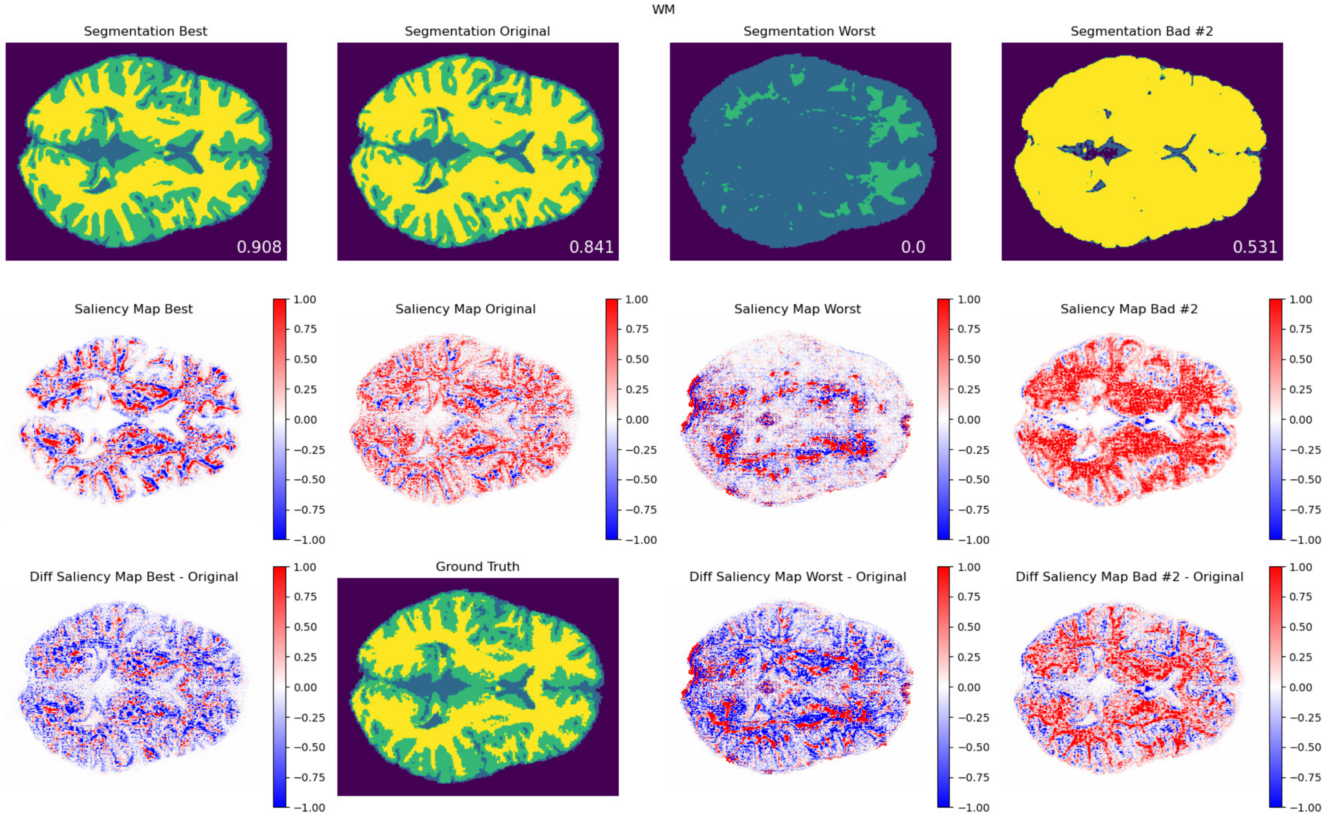

Figure S1. WM segmentation and saliency maps for the best, unmodified(Original), worst, and second bad cases in contrast modification.

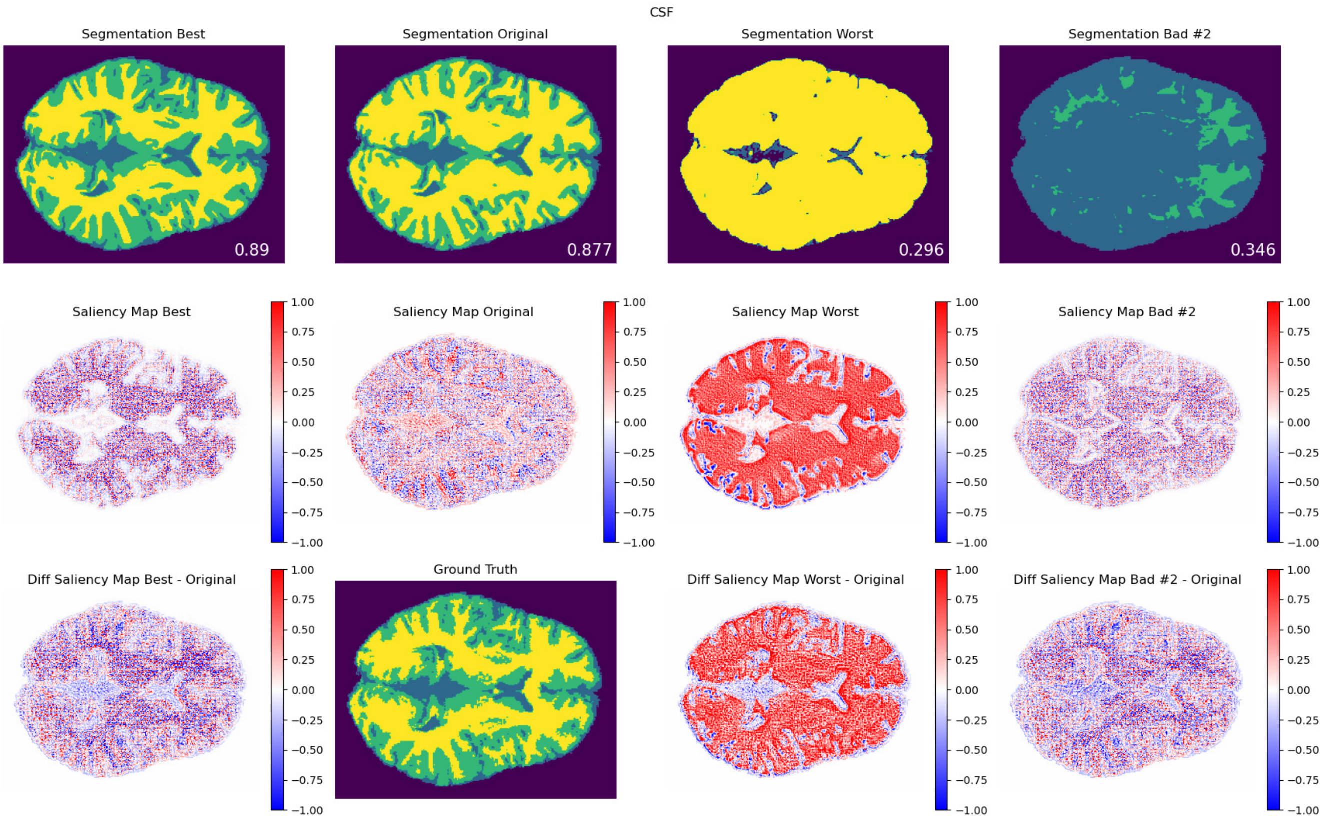

Figure S2. CSF segmentation and saliency maps for the best, unmodified(Original), worst, and second bad cases in contrast modification.

EXAMPLE SLICES OF SALIENCY MAPS OF GM AND WM IN TEXTURE MODIFICATION.

Shown in Fig. S3 and in Fig. S4, WM and CSF segmentation results (top row), saliency maps (middle row), and saliency difference maps (bottom row), for best (first column), original (second column), worst (third column), and another exemplary bad model performance (fourth column) opposite to worst in terms of the combination of modifications, for vendor TR = 500 ms; TE = 25 ms. The best, worst, and second bad cases corresponded to the blue, red-, and green-framed squares on heatmaps in Fig. 4. As a reference, the DSC values for best, original, worst, and other bad result models are included for each segmentation result. An attention shift or redistribution of pixel attribution is observed between the original, best, and worst performances.

1 ANIMATION OF SALIENCY MAP

The animation of saliency maps of gray matter change with the setting for different contrast modification pairs on the training dataset and testing dataset is also uploaded to frontiers as in additional files.

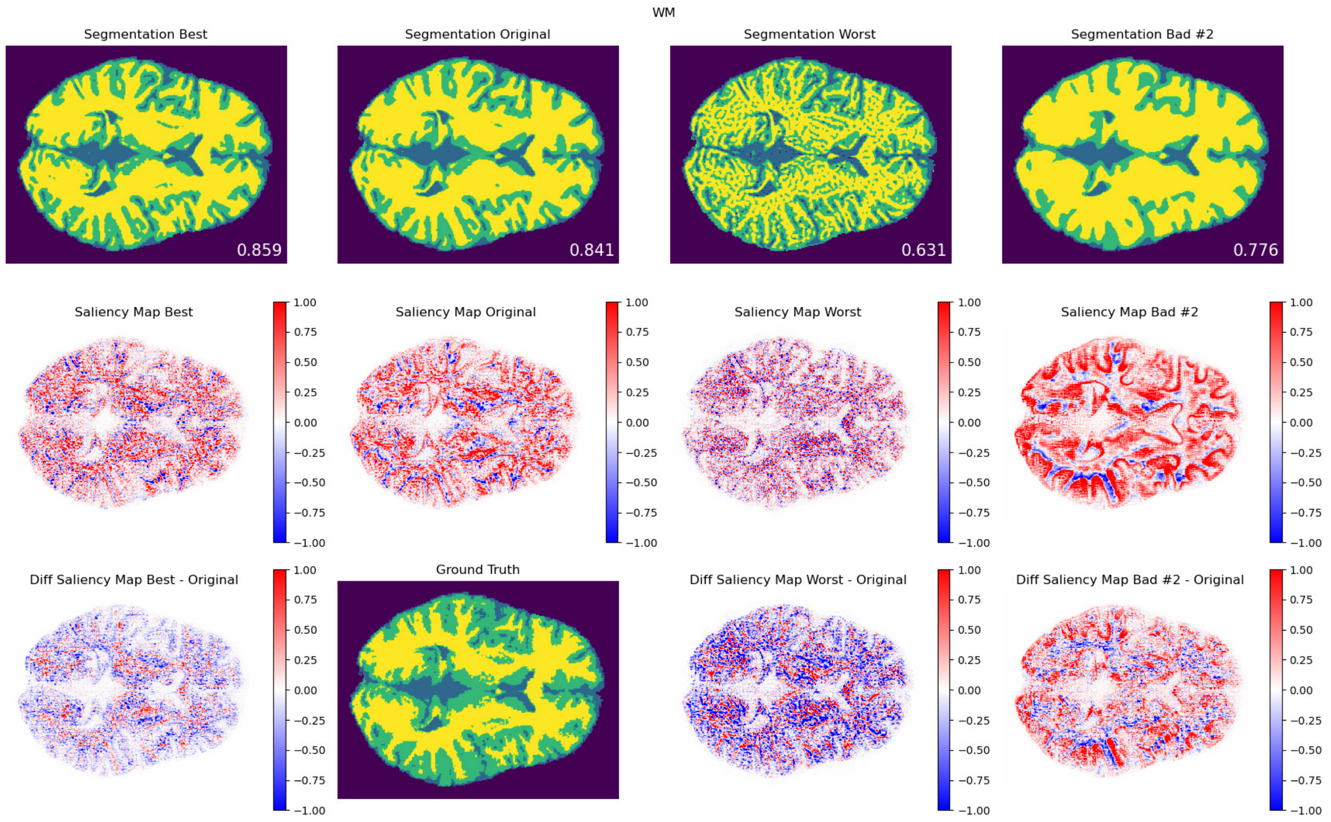

Figure S3. WM segmentation and saliency maps for the best, unmodified(Original), worst, and second bad case in texture modification.

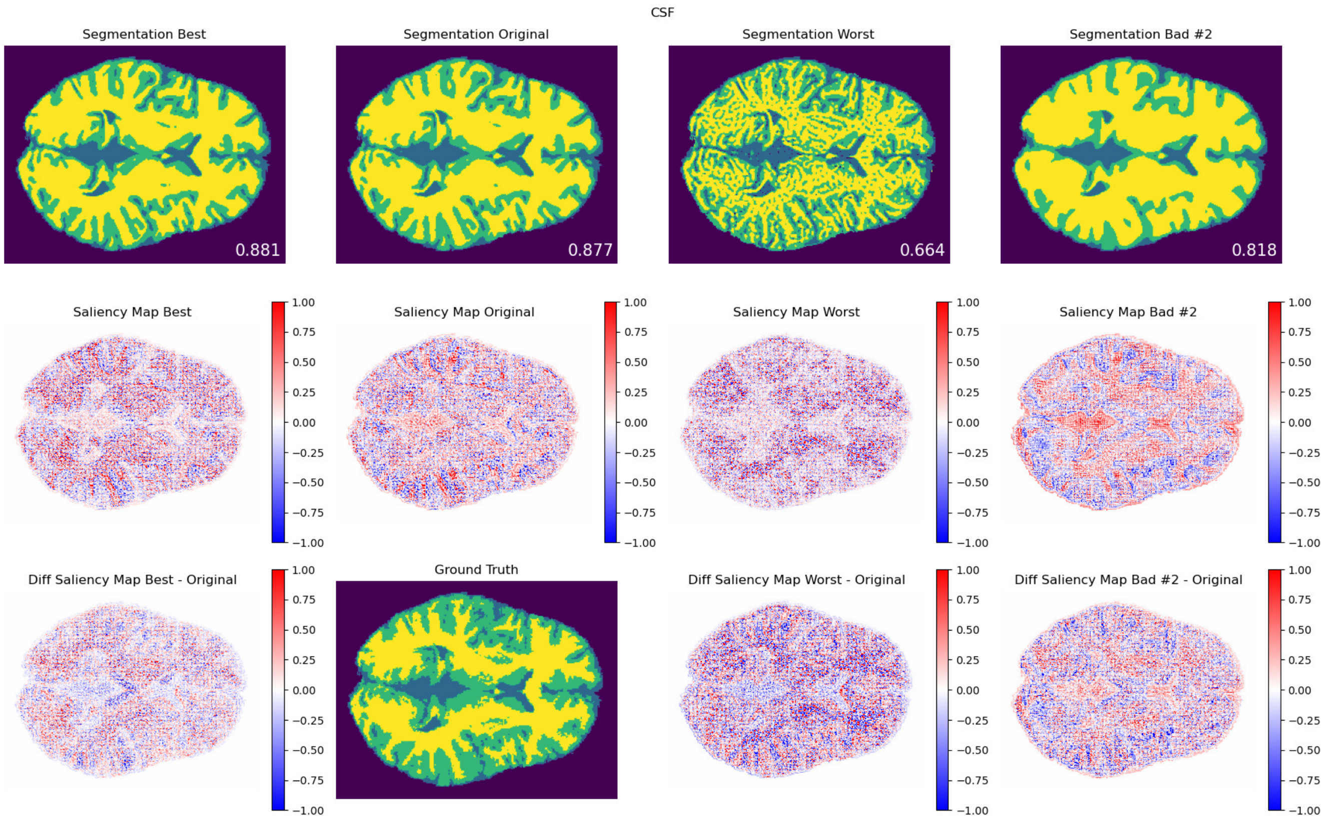

Figure S4. CSF segmentation and saliency maps for the best, unmodified(Original), worst, and second bad cases in texture modification.
